# Hybrid and Synthetic FRP Composites under Different Strain Rates: A Review

**DOI:** 10.3390/polym13193400

**Published:** 2021-10-02

**Authors:** Ali Farokhi Nejad, Mohamad Yusuf Bin Salim, Seyed Saeid Rahimian Koloor, Stanislav Petrik, Mohd Yazid Yahya, Shukur Abu Hassan, Mohd Kamal Mohd Shah

**Affiliations:** 1 Department of Mechanical and Aerospace Engineering, Politecnico di Torino, 10129 Turin, Italy; ali.farokhi@polito.it; 2 Department of Solid Mechanics, AMICI R&D Group, Tehran 1474585745, Iran; 3 School of Mechanical Engineering, Faculty of Engineering, Universiti Teknologi Malaysia, Johor Bahru 81310, Malaysia; yusufsalim.daud@gmail.com (M.Y.B.S.); shukur@utm.my (S.A.H.); 4 Institute for Nanomaterials, Advanced Technologies and Innovation (CXI), Technical University of Liberec (TUL), Studentska 2, 461 17 Liberec, Czech Republic; stanislav.petrik@tul.cz; 5 Department of Aerospace Engineering, Faculty of Engineering, Universiti Putra Malaysia, Serdang 43400, Malaysia; 6 Centre for Advanced Composite Materials (CACM), Universiti Teknologi Malaysia, Johor Bahru 81310, Malaysia; 7 Advanced Composite and Material Research Group, Faculty of Engineering, University Malaysia Sabah, Kota Kinabalu 88400, Malaysia; mkamalms@ums.edu.my

**Keywords:** hybrid composite structure, synthetic composite, impact loading, strain rate, failure mode and deformation

## Abstract

As a high-demand material, polymer matrix composites are being used in many advanced industrial applications. Due to ecological issues in the past decade, some attention has been paid to the use of natural fibers. However, using only natural fibers is not desirable for advanced applications. Therefore, hybridization of natural and synthetic fibers appears to be a good solution for the next generation of polymeric composite structures. Composite structures are normally made for various harsh operational conditions, and studies on loading rate and strain-dependency are essential in the design stage of the structures. This review aimed to highlight the different materials’ content of hybrid composites in the literature, while addressing the different methods of material characterization for various ranges of strain rates. In addition, this work covers the testing methods, possible failure, and damage mechanisms of hybrid and synthetic FRP composites. Some studies about different numerical models and analytical methods that are applicable for composite structures under different strain rates are described.

## 1. Introduction

Over the past decades, fiber-reinforced polymer (FRP) composites have found many applications in advanced industries. FRP composites are more preferable in aerospace, automotive, energy, etc., industries due to their superior characteristics, such as specific strength, good fatigue resistance, and high crashworthiness capability [1,2,3]. Industrial demands for advanced materials with enhanced and environmentally friendly material properties increase day by day. FRP composites alter wood and metals due to their lightweight, specific strength ratio, corrosion resistance, and good toughness. 

Classification of FRP composites is based on filler and base materials. The base materials are called matrices, which hold the filler material in structures. There are two main types of fibers, namely continuous and discontinuous fibers. The unidirectional, bidirectional, and 3D-woven types are classified as continuous fibers. Chopped strand mat, randomly oriented fibers, and aligned fibers are examples of discontinuous fibers. Synthetic and natural fibers are the most commonly used fibers in industry. Natural fibers reveal light density, simple productivity, and recyclability. The suitable strength, lightweight, and biodegradability of natural fibers such as kenaf, sisal, jute, coconut fiber, bamboo leaf, and flax have made polymer composites more attractive [4,5,6]. Using natural fibers as sustainable and recyclable materials has gained attention for producing natural composite structures. However, some limitations such as low strength cause them to be less applicable than synthetic fibers. Therefore, hybrid composite materials combine two or more different fibers in a single matrix to generate new material with sustainability and high strength in comparison to synthetic materials. In some studies, the hybrid composite structures were made by a combination of natural fibers, and their final strength is a challenge compared to synthetic fibers. 

From wood, plants, and waste products of the agroforestry or paper-making industry, lignocellulose fibers can be produced. Using waste materials of agroforestry and converting them to natural fibers has high added value. The use of by-products can prevent disposal in landfills and the associated contamination. Other sources for natural fibers are biological, such as air or feathers, which can be mixed with a matrix to obtain composite materials [7,8]. There are many studies that have been conducted to fabricate different kinds of natural fibers from waste by-products [5]. Recently, advanced techniques were used to improve the mechanical properties of cellulose fibers. Moreover, different chemical treatments were implemented on the natural fibers to increase their mechanical properties [6,7,8,9,10].

On the other hand, using natural fibers in hydrophobic matrices leads to weak interphase for a composite structure. It is an open topic, working to achieve interphases to provide high-strength materials under axial loading and impact. The other problem related to natural fibers is the repeatability properties from one batch to another. Several investigations have been carried out to increase the degree of certainty of material properties of natural fibers [4,10].

The other interesting ongoing topic is the application of bio-based polymer matrix composites with natural fibers. These matrices are made of oil-independent and recyclable materials. However, the price of this material is higher than that of petrochemical polymers. Recently, the use of polylactic acids (PLA) as bio-based polymers has increased. Reducing the cost and increasing the mechanical properties of bio-based polymers can be taken into consideration in future studies. In addition, using phenolic foam resin in the additive manufacturing process was reported to generate complex silicon-based composites [11]. 

The present study investigates the material constituents of synthetic and hybrid laminate composites and their behavior under impact loading. In addition, this review addresses the different methods of mechanical characterization of the composites under various ranges of strain rates. Additionally, it covers the experimental methods, possible failure modes, and damage mechanisms of various hybrid and synthetic composites. Finally, a review of studies on different numerical models and analytical methods that are applicable for such composite structures under different strain rates is presented. 

## 2. Polymer Matrix Hybrid Composites’ Contents

### 2.1. Fibers

#### 2.1.1. Synthetic Fibers

Several kinds of fibers are available in the market for producing FRP composite structures. Most of the fibers are synthetic fibers due to their strength, ease of production, long lifetime, and availability [12,13,14,15,16]. The man-made fibers are divided into two main categories, namely organic and inorganic fibers. The most well-known fibers in the world are carbon and glass fibers. However, they have many types, for example, glass fiber can be found in E-Glass, S-Glass, or other types. Synthetic fibers can be produced with different properties based on their functionalities [17]. For instance, carbon fibers can be produced with high electrical conductivity or different moisture absorption capabilities. Functional-based materials provide an opportunity for use in advanced structures, such as wind turbines and the aerospace industry. The superior mechanical strength, moisture absorption resistance, flame-resistant, and repeatable properties make them better than natural fibers to produce a commercial product. Reportedly, the range of tensile strength for E-Glass fibers was between 1.5 and 3.5 GPa, and the elastic moduli were varied between 45 and 90 GPa. These high-strength properties are higher than all-natural fibers’ properties [18]. Aramid/Kevlar is the most used organic synthesis fiber that has been used for impact application. To create bulletproof vests, armors, and military helmets, this fiber is one of the best choices, however, it is highly vulnerable to environmental attacks. Recently, some researchers have conducted studies to hybridize this fiber with carbon fibers to overcome this problem [19,20,21,22].

#### 2.1.2. Natural Fiber

The history of the first natural fibers dates back to ancient mud and straw walls. Before emerging petrochemical products, natural fibers were used in different structures, but when lightweight structures were demanded, the use of synthetic fibers increased. However, in the last decade, due to ecological effects, increasing CO_2_ emissions, recycling problems, and the high cost of synthetic fibers, natural fibers are getting more attention. The limitations of synthetic fibers can be solved by adding fillers to the composite structure. These fillers or natural fibers decrease the cost of structures regarding the strength of the composite laminate. Yorseng et al. [23] generated a bio-based hybrid composite with sisal/kenaf and bio-epoxy resin. Many mechanical tests were carried out to evaluate the endurance of the hybrid composite under weathering acceleration conditions. According to ASTM standards, a series of tests, such as tensile, axial impact, thermography, and water absorption, were performed on this hybrid composite. Jute is another type of natural fiber, which was used by Jha et al. [23,24] as the chopped strand fiber with 30% reinforcement. They combined jute with glass fiber to elevate the inferior properties of jute fibers. The hybrid jute/glass fiber composite was used to make an exhaust manifold. Their hybrid composite has better properties in terms of mechanical strength and wear resistance. They made five different hybrid composites (jute/glass/epoxy) with different fiber weight fractions, and the tensile strength and wear tests were performed on different fiber configurations to find the best composite structure. Prabhu et al. [25] have conducted a study on the hybrid jute/tea leaf/epoxy composite laminate to evaluate the quasi-static and dynamic characteristics of the hybrid structure. They performed tensile, flexural, and impact tests on different types of laminates and found the best composite structure subjected to different loading conditions. Shireesha et al. [26] investigated the effect of hybridization of jute/banana fiber/epoxy under quasi-static loading and the toughness test. They performed mechanical testing on three different hybrid composite laminates and found the best mechanical properties of the structure. In a recent study, a combination of alkali/Luffa/epoxy was investigated experimentally, where the epoxy hardener was DDM-modified by hexagonal boron nitride nanoparticles. The composite laminate includes three plies made by the hand layup method, in which the samples were used for tensile, flexural, and axial compression tests [27]. Some researchers examined the use of aerogel as the matrix reinforced by fiber [28,29,30], as a hybrid composite that benefits the fabrication of strong material with high thermal insulation. Chakraborty et al. [28] characterized and synthesized fiber-reinforced silica aerogels using hexane, trimethylchlorosilane, and ammonium fluoride through fast ambient pressure drying. The product is recommended to be very effective for firefighting system applications in extreme heat exposure conditions. Rocha et al. [29] have researched the characterization of fiber-reinforced silica aerogel for Mars exploration, to simulate Mars’ environment on the material properties. They have highlighted that the thermal feature of the composite was not affected in harsh environmental conditions, which indicated the credibility of the aerogel matrix to withstand temperature variation. Lu et al. [30] have researched simulation of the tensile behavior of a fibrous composite based on the aerogel matrix using a new multi-scale approach that considers the aerogel material in nanoscale, while the bulk composite is simulated in microscale. They have shown that the mechanical property of the composite primarily depends on the microstructural constituents of the aerogel matrix. Moreover, they have highlighted that the characteristics of the fibers have a meaningful effect on the mechanical property outcomes of the aerogel matrix. The approach was recommended to study the mechanical behavior of other aerogel materials, considered in the form of a fiber-reinforced matrix. Arthanarieswaran et al. [31] studied the hybrid material properties of a glass epoxy composite combined with different natural fibers. In this study, banana leaf and sisal fibers were used as natural fibers. A different configuration of natural fibers with glass fiber and epoxy resin was used, and nine different types of test samples were generated. To evaluate the mechanical responses of different samples, they were tested under quasi-static and impact loading, and the dynamic responses of the structure were reported. To increase the impact resistance and other mechanical properties of FRP hybrid composites, some researchers have mixed different natural fibers with synthetic fiber. In these studies, mostly the jute, banana fiber, curaua fibers, and sisal were mixed with different weight ratios, and the epoxy resin with a different kind of hardener was used as the matrix. They could improve the impact resistance of the structures and reduce the material density by around 25%. These combinations help to decrease the loss of mass as a function of temperature and water absorption. Moreover, for the curing process of hybrid laminate [32,33,34], Chee et al. [35] prepared a hybrid composite with bamboo/kenaf natural fibers and resin epoxy to evaluate the thermo-mechanical loading with different strain rates. They studied the effect of oxidation resistance and thermal stability regarding elastic modules’ degradation when the structure is subjected to different thermal loading rates. Dunne et al. characterized mechanical responses of various natural fibers generated with the Acrylonitrile Butadiene Styrene (ABS) matrix. They tested their hand layup specimen under different loading rates and evaluated the impact resistance of different hybrid composite panels [36]. Other researchers worked on the hybrid composite that was made by hemp/sisal/epoxy through the hand layup method. They analyzed static responses of the test samples with tensile, compression, and interlaminar shear strength. The other properties such as water absorption, hardness, and void density were also examined [37,38,39].

Hybrid composite structures are a combination of different fibers mixed in a single type of matrix. Many studies have been carried out on different kinds of hybrid composites [40,41,42]. Since the last decade, several studies have been conducted using one or more filters in the polymeric matrix and conventional fibers [43,44,45]. The studies were categorized in different approaches, such as experimental testing, numerical simulation, or analytical calculations. Moreover, different kinds of loading conditions and applications were considered as the main objectives of the research [46,47,48,49]. As mentioned earlier, the combination of two or more fibers in a matrix, such as ceramic, metal, or polymer, can be identified as the hybrid composite [15]. Some studies have used different polymeric matrices with fibers and fillers to fabricate hybrid composites [43,50,51]. Auto-hybrid terms have been used when the same filler and matrix are used in different laminate thicknesses. In order to fabricate a hybrid composite based on the structure, application using different fillers is a crucial matter. For example, if an E-glass/epoxy composite is hybridized with jute, the compressive capability will be increased, however, using bamboo leaf with E-glass/epoxy increases the tensile capacity of the structure. It can be said that based on each application, different fillers can be effective in structural functionality [40,52]. For example, Mansor et al. [52] developed an analytical model to select the best natural fiber between 13 fibers to fabricate a hybrid composite structure under compression and abrasion. In that study, the kenaf was the best filler when abrasive wear resistance was required. Moreover, to use an appropriate filler, the environmental condition of the hybrid composite product is important. In fact, whether the working condition is indoors or outdoors is the other key point when adding filler to the composite laminate. Therefore, a balance between environmental effects and product application should be considered. The main factors to produce hybrid composite structures are the following: weight, mechanical strength, environmental working conditions, moisture resistance, disposal ability, recyclability, and the final price. The other factor to design a hybrid composite is the capability of bonding between the filler, main fiber, and polymer matrix. In addition, the volume fraction of the synthetic fiber and filler will affect the properties of the structure that can be predicted using the rule of mixture law.

**Figure 1:**
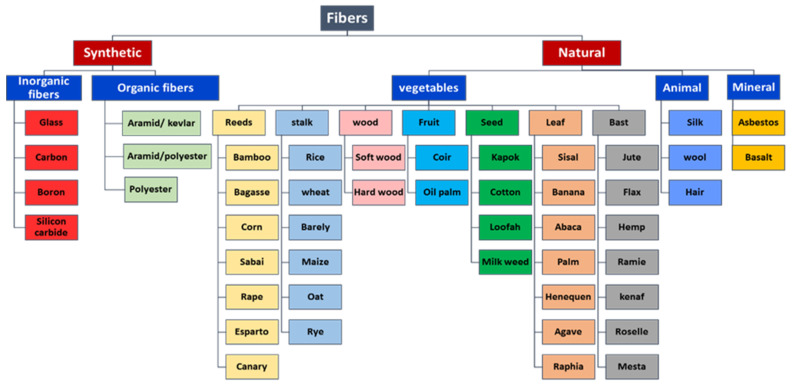
Different categories of fibers for FRP composite production.

Some limitations of natural fibers, such as dissimilarity in mechanical and physical properties, low thermal stability, large moisture absorption, and flammability, have encouraged researchers to use natural fibers in hybrid composites to overcome their limitations. They have attempted to replace synthetic fibers with natural fibers to obtain renewable, inexpensive, and biodegradable fillers. Most natural fibers are produced from animal resources, vegetable waste, and minerals. Vegetable fibers include cellulose, hemicelluloses, lignin, and pectin, which are flammable. Many types of research have been conducted to modify the fire resistance of natural fibers [53,54,55]. Figure 1 categorizes different types of fibers using fiber-reinforced composites.

### 2.2. Polymeric Resins

The common part of hybrid composites and conventional composites is the matrix. The polymeric matrix is made of resins, which create the final structure’s shape and maintain the position of the fibers. Some studies have used two different types of resins for making hybrid composites [9]. Thermoplastics and thermosets are the two main groups of resins used to produce polymer matrix composites. Many hybrid composite structures are made by thermoplastic resins. They can be molded and have a stable shape after the process. The process of using thermoplastic is to heat, liquefy, and impregnate the fibers, and then the mixture can be molded. Thermoplastics can be reheated and remolded again. The most famous thermoplastics are polypropylene (PP), polyethylene (PE), polyvinyl chloride (PVC), and polystyrene (PS). Thermoplastic resin can be used for different fabrication methods, such as resin transfer molding (RTM), sheet molding compound (SMC), pultrusion, vacuum-assisted resin transfer molding (VARTM), and hand layup, which requires less pressure [49]. The application of thermoplastics is mostly in aerospace, turbine blades, and the automotive industry due to their higher strength than thermosets [12,56].

#### 2.2.1. Thermoplastics

In order to improve the properties of the natural fibers and increase better interface bonding, some chemical or physical treatments have been applied [12,56]. Some researchers have used alkali and its combination with acetyl or saline to perform a chemical treatment. During chemical treatments, the mechanical properties of the hybrid composite can be effectively improved. To apply the physical treatment, electrical discharges such as corona and plasma were used [56]. The previous research shows that the physical or chemical treatment improves interfacial adhesion between resin and fibers, which enhances the impact resistance of the hybrid laminates. Thermoplastics can be formed by using compounding or pressing after being melted and molded [57,58,59,60]. The compound process can be performed through the screw extruder, heater, and die extrusion in a single machine. However, overheating of fiber can degrade the final product’s properties. Therefore, polymer selection is the key point to save the properties of the natural fiber due to overheating degradation. To overcome this problem, polyolefins such as polyethylene and polypropylene can be used to protect natural fibers. Most of these polyolefins are melted below 200 °C, which is appropriate to produce the hybrid composite with natural fibers [46,48,61,62]. Some studies have been conducted to evaluate the interface bonding on polyolefin and hydrophilic natural fibers, and they reported weak interfacial bonding [62,63]. To improve the interface properties, some reinforced natural-based polymers, including polylactic acid (PLA), poly (butylene succinate-co-lactate) (PBSL), thermoplastic starch (TPS), poly hydroxyl alkanets (PHA), and many others, have been proposed [57,58,59]. Some research results show the poor strength of bonding in natural fiber composites. Therefore, chemical treatment such as fiber impregnation by silane can significantly improve the quality of the final product [57,59,63,64]. Some studies have been conducted using PLA, which is a good competitor for synthetic resins, such as polypropylene and polystyrene [63]. Yauri et al. and Chen et al. [65,66] have used chopped strand mat, unidirectional and woven within melted PLA through the compression process. They obtained good outcomes with this hybrid composite under different mechanical tests. In some cases, the resin is dissolved in a solvent such as water and mixed with the filler to obtain suitable distribution along with the content. Edhirej et al. [67] generated a hybrid composite using a combination of cassava starch, fructose, and reinforced with sugar palm fiber, as well as water as a suitable solvent. However, due to the price and non-solubility of most polymers, this method is not applicable for commercial products. 

#### 2.2.2. Thermosets

Thermoset resins are well-known polymers with 3D cross-linked networks with a curing process. The most used thermosets are epoxy resins, which are available in different categories. Different epoxies have variations in their viscosities, which helps to choose the suitable epoxy type based on their application. They have lower shrinkage than other types of thermosets. In most advanced applications such as aerospace, light turbine blades, or the automotive industry, the epoxy resin with a hardener agent or curing technique has been used [68,69,70,71,72]. Recently, the hybrid sintered carbon/basalt epoxy composite was used as the friction material in the shifting mechanism to reduce the synchronization time in a transmission system [73,74]. 

Moreover, epoxy resins can be used in almost all fabrication methods [14,75,76,77,78]. The other prominent thermoset resins can be identified as polyester, phenolic resins, polyurethanes, acrylics, alkyds, furans, polyamides, and vinyl esters. More attention has been paid to the epoxy and polyester resins to produce the hybrid composites [79]. A similar production method with epoxy resin can be applied to the polyester resin regarding hybrid composite fabrication. Moreover, the polyester resin and its hardener agents are highly effective on the mechanical properties of the final composite product. The long curing time is the weakness of using thermosets, and they are not able to directly recycle. A recent study has used ground-cured thermosets and used recycled thermosets again as the filler. Many studies modified the toughness of thermosets by increasing the process temperature. The maximum temperature will not affect the properties of the natural fiber. Different production methods were applied to use thermosets, such as impregnated fibers with resin and hand layup, vacuum bag resin transfer molding (RTM), and vacuum-assisted transfer molding (VARTM). For advanced applications such as aerospace and automotive industries, VARTM was mostly implemented [80,81,82,83]. This method has a low preparation cost and also has low unstable organic compounds’ emissions. Hybrid thermoset composites were fabricated on a laboratory scale by the hand layup method due to the ease of fabrication and lower cost [84,85]. 

## 3. Hybrid and Synthetic FRP Composites under Different Loading

Many studies have been conducted on polymeric composite structures. Besides content materials in a hybrid composite, the dynamic responses of fabricated composites are highly necessary to understand. The strain rate effect on different hybrid composites is a key point to select a composite laminate structure for advanced industrial applications [86,87,88,89,90]. The composite products that are subjected to the impact loading are required to be studied in different approaches. The responses in quasi-static loading, low-impact, high-impact, and post-failure analysis have been conducted on synthetic composite structures. Although the majority of applications in the composite structures market are for synthetic composites, minor attention has been paid to the hybrid composites under dynamic loading. Hybrid composites have some content in common with conventional composites, however, the natural fiber or resins in the content of hybrid composites will change the microstructure of the hybrid composite, which is necessary to study. Reviewing the existing information about the impact resistance of different hybrid composites is the second objective of this study.

Previous studies have conducted experimental tests of different strain rates’ effects on some polymers. Kolsky, Davies, and Hunter [91,92] carried out adequate studies on the stress-strain relationship of some polymeric composites under the range of strain rate between 10^−4^ and 10^5^ s^−1^ [93,94,95]. Many kinds of polymer structures have time-dependent behavior that is proven by their rate-dependent elastic modulus, yield point, and plastic behavior. Moreover, varying temperature and rate-dependent behavior affect the physical shape, from rubber state to ductile and brittle [96,97,98,99,100,101,102]. Strain hardening phenomena have been reported for hyperplastic materials and the matrix with large deformation behavior [103]. To observe the effect of strain rate on tensile modulus, the time-temperature responses were superimposed. Moreover, in other studies, strain rate dependency on yield stress was considered by superposition of time-temperature data [104,105,106,107,108,109]. In those studies, the linear mapping of temperature and strain rate was applied, and it illustrated that the lower β transition is rate-dependent in high-strength glassy polymers. Based on the polymer microstructure, many rate-dependent constitutive models have been developed [109,110,111,112,113,114]. To simulate the rate dependency of different materials, commercial codes and software were used. The first model was a one-dimensional model proposed by Ree and Eyring [115]. Furthermore, this theory has been modified for 3D models with a dependency on strain rate, temperature, and pressure [116,117]. In order to characterize composite materials under different loading rates, many experimental tests have been proposed. Increasingly, these tests are identified as the guidelines for different tests and various applications. 

Many empirical models have been proposed to simulate the behavior of the composite matrix, however, to modify these models, an adequate dataset is needed [111,118,119,120,121,122,123]. In hybrid composites, the structures mostly have anisotropic behavior, and this increases the uncertainty of the proposed models. Therefore, a sufficient study on existing experimental tests and overall responses of each category is necessary to help researchers to gain a better understanding when developing their analytical and numerical models.

### 3.1. Low Strain Rate Experiments

**Figure 2:**
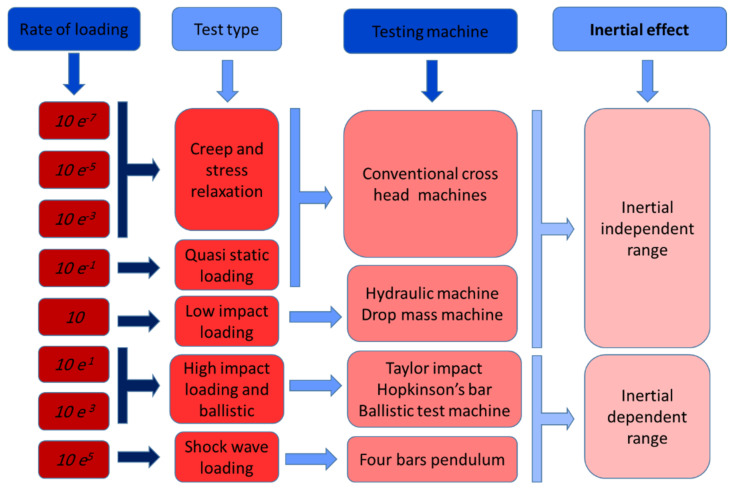
Experimental tests for composite material characterization and variation of loading rates.

The lowest rate of testing that has been applied to the composite materials is the creep machine. In terms of composite material, the creep test in the presence of moisture is a common test that is applied on natural, hybrid, and synthetic fibers, with the strain rate between 1 × 10^−7^ s^−1^ and 1 × 10^−3^ s^−1^. Some studies have been carried out to evaluate the moisture absorption under the low strain rate of FRP composites [88,124,125]. Figure 2 shows the variation of loading rates and their range of interest.

A common test that is applied to all kinds of composites is the quasi-static test. To perform a quasi-static test, normally, the universal testing machine (UTM) is used. This machine is used for tensile and compression tests directly. Moreover, the machine can be used for the flexural bending test or the shear test by using various tools. These kinds of machines were equipped with servo-hydraulic actuators with a speed of 1–25 mm/min. To perform cyclic loading, the servo-hydraulic machine can provide the load frequency between 2 and 100 Hz, then the approximation between the tension and compression strain rate of the test can be calculated by Equation (1) [126]:

(1)  ε˙≈ΔεΔt=ε014f=4fε0

where f is test frequency and $\varepsilon_{0}$ is the strain amplitude applied on the testing machine. Many studies have been conducted on this range of strain rates [127,128,129,130,131,132].

### 3.2. Medium Strain Rate Experiments

**Figure 3:**
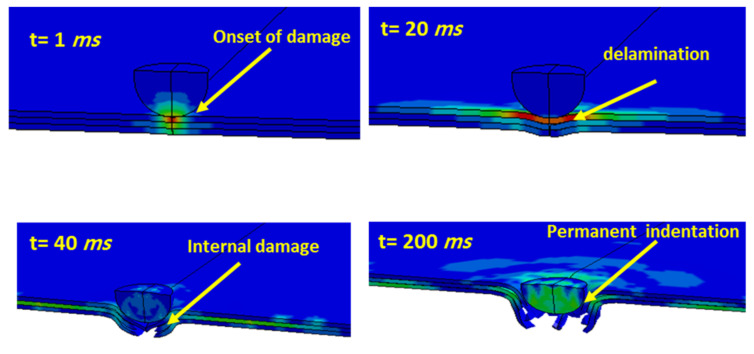
A damaged hybrid composite laminate under low-impact loading.

The range of strain rate for low-velocity impact is normally between 1 and 5 s^−1^, which is an important range for all materials to characterize. From 5 s^−1^ to 2 × 10^10^ s^−1^ is categorized as the high-velocity impact. Figure 3 shows a damaged hybrid composite laminate under low-impact loading. The penetration and internal damage can be seen in the figure.

The intermediate strain rate experiments are generally carried out by hydraulic high-speed machines. Some studies characterizing the FRP composites under impact loading used the dropping weight tests as well [127,133,134,135,136]. Other experiments have been conducted to extract dynamic responses of polymeric composite structures, e.g., flywheel cam system and pneumatic expanding ring [137,138,139,140].

**Figure 4:**
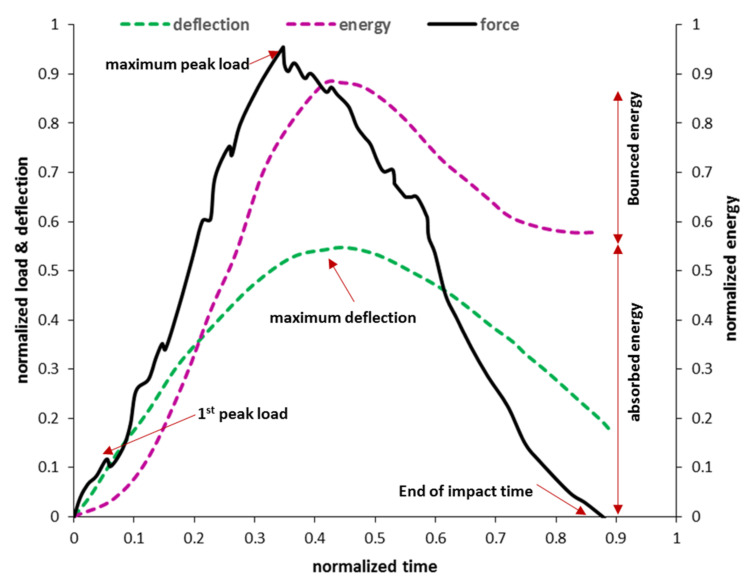
A schematic plot for load, deflection, and energy as a function of time [148].

The first impact indicators of the FRP composites are energy, force, and displacement, which can be extracted as the function of time. Throughout the impact test, the stress wave fluctuates, and it can be seen on the force-time graph. The peak point of this graph represents the maximum impact resistance of the structure, and after this point, the composite structure stiffness degrades rapidly. Figure 4 shows a schematic plot for load, deflection, and energy as a function of time. The combination of bounced energy and absorbed energy is the total energy applied on the impact test. It can be said that these two sources of energy are the main terms of energy in the impact test, and by ignoring other energy dissipation sources, the external work is almost equal to the summation of elastic energy and absorbed energy. The maximum permanent deflection is directly affected by the magnitude of energy absorption. Several studies have been conducted to evaluate impact indicators under low- and high-velocity impact loading [90,141,142,143,144]. In addition, recently, some studies have investigated the impact resistance of hybrid composites under impact loading [145,146].

Based on ASTM standard D7136, the maximum deflection over the impact test can be calculated as a function of time from Equation (2) [147]:

(2) $$\delta \left(t\right)=\;\delta \left(i\right)+V_{i}\;t+\frac{{\mathrm{gt}}^{2}}{2}+\int_{0}^{t}\left(\int_{0}^{t}\left(\frac{f\left(t\right)}{m}\right)\mathrm{dt}\right)\mathrm{dt}$$

where $V_{i}$ and $\delta \left(i\right)$ are the initial velocity and initial deflection respectively, and $f\left(t\right)$ is the reaction force in a specific time increment. From the same standard, the energy absorption of a composite plate under impact can be calculated as Equation (3) [147]:

(3) $$E\left(t\right)=m\left(V_{i}-V^{t}\left(t\right)\right)+\mathrm{mg}\delta$$

**Figure 5:**
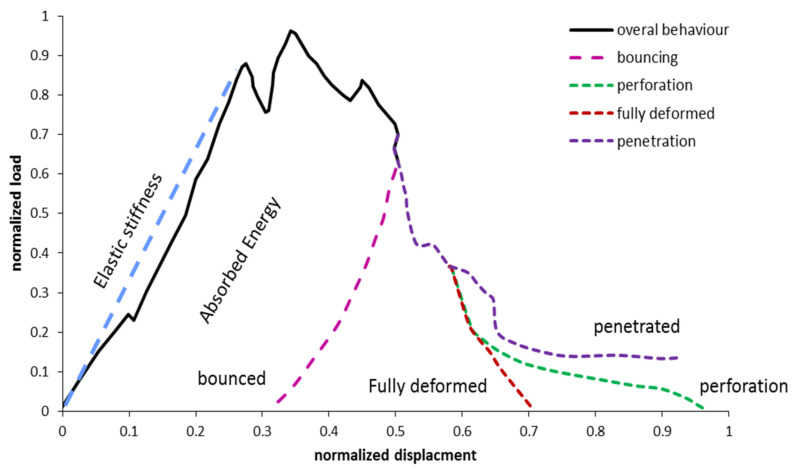
A typical load-deformation relation of an FRP composite plate under low-impact loading.

Figure 5 represents a typical load-displacement curve of an FRP composite plate under low-impact loading. The area under the curve presents the energy absorption value. Different responses of composite plates under low-velocity impactors can be seen in Figure 5. The bounced indenter, penetrated, fully deformed structure and perforated plate are the different damage mechanisms that can be observed in the low strain rate test. The first slope of the graph expresses the elastic bending stiffness of the plate.

**Figure 6:**
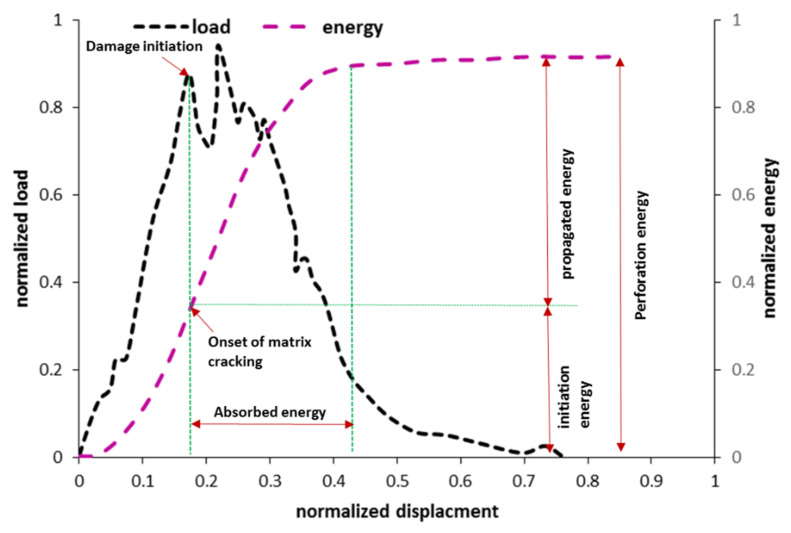
A typical plot of the load-displacement and energy-displacement curves for general FRP composites under impact loading.

Figure 6 indicates a typical plot of the load-displacement and energy-displacement curves for general FRP composites under impact loading. At the first peak load, the damage initiates with matrix cracking. The intersection of this point with the energy-displacement curve identifies the border between damage initiation and damage propagation. The damage initiation point to the energy propagated region can be called the energy absorption area.

### 3.3. High Strain Rate Experiments

**Figure 7:**
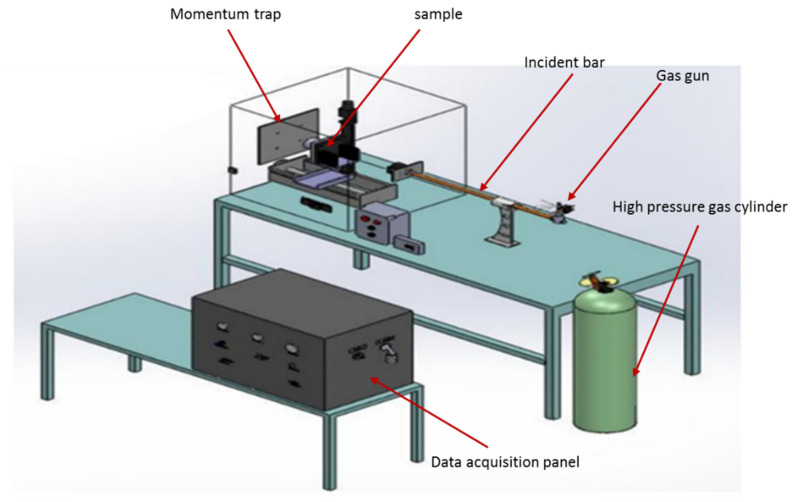
A schematic view of a Split Hopkinson Pressure Bar (SHPB) machine.

Advanced technologies and industries need to improve the impact resistance of their products subjected to high strain rates. Hence, several studies have been conducted to investigate the effect of high strain rates on various types of composites. Different testing methods were used to characterize the high strain rate properties of composite structures. For material characterization in the range of 5 × 10^2^ to 1 × 10^4^ s^−1^, the Split Hopkinson Pressure Bar (SHPB) machine is the most common testing method. During this test, the sample is clamped between two slender bars as the input and output tools. The strain gauges or Photon Doppler Velocimetry (PDV) were implemented on the machine and an incident wave and transmitted signal were obtained from the test. The shock wave is generated from a high-pressure gas or electromagnetic field, and by changing the impedance from the input bar to the output bar due to the material properties of the sample, the reflected signal is measured. Figure 7 shows a schematic view of an SHPB setup. Some studies have been performed to characterized high strain rate properties of the polymeric matrix and composite materials [149,150,151,152].

**Figure 8:**
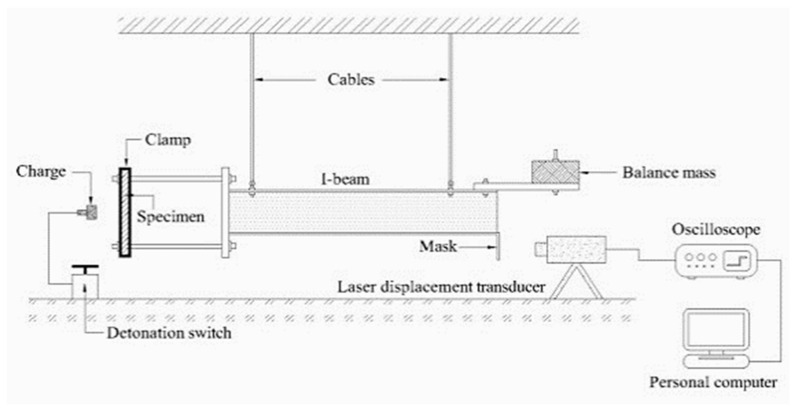
A schematic view of a four-pendulum bar mechanism for material characterization of a fiber metal laminate composite under impulsive loading [153].

The other test to characterize the high strain rate response of a composite plate against impulsive load or a shock wave is the four-pendulum bar mechanism. The strain rate for this test is between 1 × 10^5^ and 1 × 10^6^. Figure 8 represents the schematic view of a four-pendulum bar mechanism for material characterization of a fiber metal laminate composite under impulsive loading [153,154]. 

Some studies have been conducted on high strain rate analysis of shock waves for composite structures. However, mostly, the polymeric composite structures were used as the core materials, as well as increasing the moment of inertia to make lightweight and impact-resistant structures. Generally, this kind of structure is intensified with two or more metallic face sheets [155].

### 3.4. Failure Modes in FRP Composites under Impact Loading

The failure modes for FRP composites under impact loading were reported in some studies. The main damage mechanisms at different strain rates that are measured in micro- to macro-levels include micro-matrix cracking, fiber and matrix deboning, delamination, matrix damage-induced delamination, fiber breakage, intralaminar shear, in-plane shear, progressive damage, tulip damage, back face sheet pealing, structural failure, and global excessive deformation to rupture phenomena [78,114,119,123,153,156]. Table 1 categorizes different failure modes for synthetic FRP composites [157].

In hybrid composites, the main sources of damage are fiber debonding, delamination, matrix failure, fiber failure, and in-plane shear. Several studies have been conducted on the strain rate effect of hybrid composites. Table 2 represents the reported studies on the experimental tests of the low- and high-impact velocity for hybrid FRP composites.

## 4. Numerical and Analytical Models

Hybrid and synthetic composites have complex behavior under different strain rates. In general, the implementation of an experimental test of complex materials at different micro- to macro-scales is always time-consuming and expensive. Therefore, the numerical and analytical models are applied as the crucial methods to study the responses of complex materials and structures [75,201,202,203]. Several studies have developed different analytical models to simulate a low strain rate to a high strain rate range of hybrid composites. Many rate-dependent and rate-independent analytical models were presented for different composite materials [89,204]. Some of these models were developed based on damage and failure of composite contents, i.e., matrix and fibers [205,206,207,208,209,210,211,212,213,214,215]. Table 3 shows the failure criteria that are applied for FRP composite laminates regarding strain rate dependency. The theories were developed over time and many models are available for different strain rates. However, existing models in dynamic loading can be more developed in terms of failure criteria regarding the thickness effect and delamination phenomena.

The theory of continuum damage mechanics, cohesive interface damage, progressive collapse, and different failure criteria were developed in finite element codes and were implemented in open source and commercial software. Nowadays, a wide range of software can simulate the dynamic responses of composite structures. Generally, commercial software such as ABAQUS and ANSYS were used to model the behavior of different structures in the range of strain rates between 1 × 10^−5^ s^−1^ and 1 × 10^2^ s^−1^. However, LS-DYNA and AUTODYN are mostly used for higher strain rates (1 × 10^2^ s^−1^ to 1 × 10^6^ s^−1^) [192,216]. Table 4 presents recent studies on the modeling activities of rate-dependent theoretical models developed in FE software.

## 5. Concluding Remarks

This study reviewed several studies of polymeric fiber-reinforced composites under different loading strain rates. Nowadays, hybrid composites are in demand due to their eco-friendliness and recycling ability. The natural fibers and synthetic fibers were comprehensively reported in this work. Moreover, the possible polymers used to fabricate the matrix with synthetic and hybrid composites were reported. Different composites have various responses under different impact velocities. In this study, material characterization methods for different strain rates were reported. Moreover, a brief introduction on damage and failure mechanisms for composite structures regarding rate dependency has been reported. The important analytical models and numerical approaches used in previous literature have been reported.

### Existing Challenges

Based on the current research, there are still many challenging topics to study in the future, as presented below:

- Less attention has been paid to hybrid structures under high-impact loading.
- Hybrid structures should take advantage of modern fabrication techniques to reduce the cost, provide repeatability, and increase mechanical properties.
- The most critical failure mode in hybrid and natural fiber composites is related to the interface bonding and interphase region, which can be intensified by using advanced preparation and fabrication techniques.

## Figures and Tables

**Table 1:** Different failure modes for synthetic FRP composites.

Materials	Loading Type	Fracture Mode	Reference
CFRP/epoxy	Low/High-impact load	Mode II, deboning, delamination	[87,158,159,160]
GFRP/epoxy	Quasi-static	progressive damage	[161,162,163,164,165,166]
GFRP/epoxy	Low-impact load	matrix cracking, Mode II, debonding	[167,168,169,170]
Graphite/epoxy	Ballistic load	fiber breakage, tulip damage, back face sheet	[171,172,173,174]
GFRP/polyester	Quasi-static	fiber breakage, progressive damage	[175,176]
CFRP/epoxy	Quasi-static	progressive damage, fiber and matrix deboning	[177,178,179]
S2 GFRP/epoxy	Quasi-static	progressive damage	[180,181]
CFRP/PEEK	High-impact load	tulip damage, back face sheet pealing, tulip mode	[182]
PPS/GFRP	Low-impact load	fiber breakage, global deformation, Mode II	[112]
GFRP/ Polyamide	Quasi-static	fiber and matrix deboning, fiber breakage	[183]
GFRP/ Polyethylene	Quasi-static	fiber and matrix deboning, Mode II	[184]

**Table 2:** Reported studies on the experimental tests of the low- and high-impact velocity for hybrid FRP composites.

Materials	Loading Type	Reference
Kevlar/CFRP/GFRP	High-impact load	[181,185]
Glass-Polypropylene, Glass-Nylon fiber	Low-impact load	[186]
Cotton-Glass fiber	Low-impact load	[187]
Glass-Carbon fiber	Low-impact load	[188]
Kevlar-Glass fiber	Low-impact load	[189]
Kenaf-Kevlar fiber	Low-impact load	[190]
Glass-Hemp-Basalt-Flax fiber	High-impact load	[191]
Glass-Carbon fiber	High-impact load	[192,193]
Glass-Kenaf fiber	Low-impact load	[194]
Kevlar-Kenaf fiber	High-impact load	[21]
Basalt-Carbon fiber	Low-impact load	[195]
Carbon-Basalt fiber	High-impact load	[146]
Glass-Kenaf fiber	Low-impact load	[79]
Carbon-Flax fiber	Low-impact load	[196]
Kevlar-Basalt fiber	Low-impact load	[197]
Glass-Carbon fiber	Low-impact load	[198]
Areca-Eucalyptus fiber	Low-impact load	[199]
Flax-Basalt fiber	Low-impact load	[200]

**Table 3:** Failure criteria for FRP composite laminates regarding strain rate dependency.

Author	Criteria	Year	Strain Rate	Reference
Tsai and Wu	Tsai-Wu	1971	Rate-independent	[205]
Tsai-Hill	Tsai-Hill	1998	Rate-independent	[206]
Aziz-Tsai	Aziz-Tsai	1965	Rate-independent	[207]
Hoffman and Chamois	Hoffman-Chamois	1969	Rate-independent	[208,209]
Tessler, A	Zigzag theory	2009	Rate-independent	[210]
Foulk, J. W.	Cohesive zone model	2000	Rate-independent	[211]
Hashin	Hashine damage	1973–1980	Low strain rate	[212,213]
Esposito L	Delamination failure	2010	Low strain rate	[214]
Cowper-Symonds	Cowper-Symonds	1957	Low strain rate	[215]
Yen-Caiazzo	Yen-Caiazzo	2002	High strain rate	[216]
De Luca et al.	High strain rate damage model	2017	High strain rate	[217]
NU-Daniel	Dynamic loading yield criteria	2016	High strain rate	[218]

**Table 4:** Rate-dependent theoretical models developed in FE software.

Material Type	Modeling Method	Objective of Analysis	Strain Rate	Reference
GFRP/epoxy	analytical	Failure analysis	Low strain rate	[219,220]
CFRP/epoxy	FE	Damage evaluation	Low strain rate	[221,222]
Graphite/epoxy	FE	Damage evaluation	Low strain rate	[113]
Carbon/glass/Kevlar/epoxy	FE	Dynamic response	High strain rate	[223]
CFRP/PEEK/epoxy	FE	Damage evaluation	High strain rate	[224]
GFRP/epoxy	FE	Damage evaluation	High strain rate	[225]
Kevlar/polypropylene	FE	Dynamic response	High strain rate	[226]
GFRP/polyester	analytical	Global deformation	Low strain rate	[227]
FRP laminate	analytical	Stress intensity factor	Low strain rate	[175,176]
GFRP laminate	analytical	Mechanical properties	Low strain rate	[161,162,163,164,165,166]
Glass laminate	analytical	Blast analysis	High strain rate	[159,160]
Polyvinyl butyral/glass	FE	Dynamic response	Low strain rate	[228,229]
GFRP laminate	FE	Blast Failure analysis	High strain rate	[230]
GFRP laminate	FE	Crack propagation	High strain rate	[231]
FRP composites	analytical	Failure criteria	High strain rate	[210,212]

## Data Availability

The data presented in this study are available on request from the corresponding author.
